# Supplementary material for: Pyruvate Dehydrogenase Kinase 1 inhibition mediated oxidative phosphorylation enhancement in cartilage promotes osteoarthritis progression
Source: BMC Musculoskelet Disord. 2023 Jul 20;24:597. doi: 10.1186/s12891-023-06585-6 (PMC10357736; doi:10.1186/s12891-023-06585-6)
Supplement: Supplementary file 1 — Additional file 1. [file 12891_2023_6585_MOESM1_ESM.zip › BP.Gsea.1653623667859/GOBP_PROTEIN_N_LINKED_GLYCOSYLATION.html]

Details for gene set GOBP\_PROTEIN\_N\_LINKED\_GLYCOSYLATION[GSEA]

|  || Dataset | 55457\_collapsed\_to\_symbols.cls.cls #OA\_versus\_normal.cls.cls #OA\_versus\_normal\_repos |
| Phenotype | cls.cls#OA\_versus\_normal\_repos |
| Upregulated in class | OA |
| GeneSet | GOBP\_PROTEIN\_N\_LINKED\_GLYCOSYLATION |
| Enrichment Score (ES) | 0.44344738 |
| Normalized Enrichment Score (NES) | 1.824548 |
| Nominal p-value | 0.0 |
| FDR q-value | 0.06914169 |
| FWER p-Value | 0.95 |
Table: GSEA Results Summary

  

Fig 1: Enrichment plot: GOBP\_PROTEIN\_N\_LINKED\_GLYCOSYLATION      
 Profile of the Running ES Score & Positions of GeneSet Members on the Rank Ordered List

  

| SYMBOL | TITLE | RANK IN GENE LIST | RANK METRIC SCORE | RUNNING ES | CORE ENRICHMENT || 1 | TMEM258 | transmembrane protein 258 [Source:HGNC Symbol;Acc:HGNC:1164] | 123 | 0.884 | 0.0440 | Yes |
| 2 | EXT2 | exostosin glycosyltransferase 2 [Source:HGNC Symbol;Acc:HGNC:3513] | 170 | 0.838 | 0.0911 | Yes |
| 3 | MGAT4C | MGAT4 family member C [Source:HGNC Symbol;Acc:HGNC:30871] | 234 | 0.796 | 0.1344 | Yes |
| 4 | DDOST | dolichyl-diphosphooligosaccharide--protein glycosyltransferase non-catalytic subunit [Source:HGNC Symbol;Acc:HGNC:2728] | 428 | 0.695 | 0.1615 | Yes |
| 5 | MGAT4A | "alpha-1,3-mannosyl-glycoprotein 4-beta-N-acetylglucosaminyltransferase A [Source:HGNC Symbol;Acc:HGNC:7047]" | 657 | 0.604 | 0.1803 | Yes |
| 6 | DOLK | dolichol kinase [Source:HGNC Symbol;Acc:HGNC:23406] | 683 | 0.598 | 0.2145 | Yes |
| 7 | ALG6 | "ALG6 alpha-1,3-glucosyltransferase [Source:HGNC Symbol;Acc:HGNC:23157]" | 696 | 0.595 | 0.2496 | Yes |
| 8 | DAD1 | defender against cell death 1 [Source:HGNC Symbol;Acc:HGNC:2664] | 737 | 0.585 | 0.2819 | Yes |
| 9 | ALG8 | "ALG8 alpha-1,3-glucosyltransferase [Source:HGNC Symbol;Acc:HGNC:23161]" | 900 | 0.543 | 0.3021 | Yes |
| 10 | ALG5 | ALG5 dolichyl-phosphate beta-glucosyltransferase [Source:HGNC Symbol;Acc:HGNC:20266] | 945 | 0.529 | 0.3308 | Yes |
| 11 | MAN1C1 | mannosidase alpha class 1C member 1 [Source:HGNC Symbol;Acc:HGNC:19080] | 1275 | 0.463 | 0.3332 | Yes |
| 12 | RPN2 | ribophorin II [Source:HGNC Symbol;Acc:HGNC:10382] | 1294 | 0.459 | 0.3596 | Yes |
| 13 | B4GALT7 | "beta-1,4-galactosyltransferase 7 [Source:HGNC Symbol;Acc:HGNC:930]" | 1411 | 0.442 | 0.3773 | Yes |
| 14 | UBE2J1 | ubiquitin conjugating enzyme E2 J1 [Source:HGNC Symbol;Acc:HGNC:17598] | 1433 | 0.438 | 0.4022 | Yes |
| 15 | ALG9 | "ALG9 alpha-1,2-mannosyltransferase [Source:HGNC Symbol;Acc:HGNC:15672]" | 1487 | 0.431 | 0.4241 | Yes |
| 16 | FUT8 | fucosyltransferase 8 [Source:HGNC Symbol;Acc:HGNC:4019] | 1565 | 0.418 | 0.4434 | Yes |
| 17 | MLEC | malectin [Source:HGNC Symbol;Acc:HGNC:28973] | 2436 | 0.310 | 0.3945 | No |
| 18 | ST6GAL1 | "ST6 beta-galactoside alpha-2,6-sialyltransferase 1 [Source:HGNC Symbol;Acc:HGNC:10860]" | 3502 | 0.202 | 0.3238 | No |
| 19 | ALG12 | "ALG12 alpha-1,6-mannosyltransferase [Source:HGNC Symbol;Acc:HGNC:19358]" | 3723 | 0.183 | 0.3178 | No |
| 20 | MAGT1 | magnesium transporter 1 [Source:HGNC Symbol;Acc:HGNC:28880] | 3920 | 0.163 | 0.3124 | No |
| 21 | TUSC3 | tumor suppressor candidate 3 [Source:HGNC Symbol;Acc:HGNC:30242] | 3940 | 0.161 | 0.3207 | No |
| 22 | ALG3 | "ALG3 alpha-1,3- mannosyltransferase [Source:HGNC Symbol;Acc:HGNC:23056]" | 4096 | 0.146 | 0.3175 | No |
| 23 | PMM1 | phosphomannomutase 1 [Source:HGNC Symbol;Acc:HGNC:9114] | 4238 | 0.134 | 0.3146 | No |
| 24 | DHDDS | dehydrodolichyl diphosphate synthase subunit [Source:HGNC Symbol;Acc:HGNC:20603] | 4356 | 0.124 | 0.3130 | No |
| 25 | STT3A | STT3 oligosaccharyltransferase complex catalytic subunit A [Source:HGNC Symbol;Acc:HGNC:6172] | 4618 | 0.103 | 0.2989 | No |
| 26 | MGAT5 | "alpha-1,6-mannosylglycoprotein 6-beta-N-acetylglucosaminyltransferase [Source:HGNC Symbol;Acc:HGNC:7049]" | 5086 | 0.066 | 0.2665 | No |
| 27 | PGM3 | phosphoglucomutase 3 [Source:HGNC Symbol;Acc:HGNC:8907] | 5290 | 0.049 | 0.2537 | No |
| 28 | MGAT2 | "alpha-1,6-mannosyl-glycoprotein 2-beta-N-acetylglucosaminyltransferase [Source:HGNC Symbol;Acc:HGNC:7045]" | 5317 | 0.046 | 0.2545 | No |
| 29 | DOLPP1 | dolichyldiphosphatase 1 [Source:HGNC Symbol;Acc:HGNC:29565] | 5369 | 0.043 | 0.2531 | No |
| 30 | MGAT1 | "alpha-1,3-mannosyl-glycoprotein 2-beta-N-acetylglucosaminyltransferase [Source:HGNC Symbol;Acc:HGNC:7044]" | 5407 | 0.040 | 0.2526 | No |
| 31 | GFPT1 | glutamine--fructose-6-phosphate transaminase 1 [Source:HGNC Symbol;Acc:HGNC:4241] | 5847 | 0.005 | 0.2187 | No |
| 32 | GFPT2 | glutamine-fructose-6-phosphate transaminase 2 [Source:HGNC Symbol;Acc:HGNC:4242] | 5849 | 0.005 | 0.2190 | No |
| 33 | MGAT4B | "alpha-1,3-mannosyl-glycoprotein 4-beta-N-acetylglucosaminyltransferase B [Source:HGNC Symbol;Acc:HGNC:7048]" | 5956 | -0.002 | 0.2108 | No |
| 34 | NUS1 | NUS1 dehydrodolichyl diphosphate synthase subunit [Source:HGNC Symbol;Acc:HGNC:21042] | 6415 | -0.037 | 0.1774 | No |
| 35 | SLC39A8 | solute carrier family 39 member 8 [Source:HGNC Symbol;Acc:HGNC:20862] | 6450 | -0.039 | 0.1771 | No |
| 36 | GORASP1 | golgi reassembly stacking protein 1 [Source:HGNC Symbol;Acc:HGNC:16769] | 6526 | -0.044 | 0.1739 | No |
| 37 | RPN1 | ribophorin I [Source:HGNC Symbol;Acc:HGNC:10381] | 6618 | -0.051 | 0.1699 | No |
| 38 | SRD5A3 | steroid 5 alpha-reductase 3 [Source:HGNC Symbol;Acc:HGNC:25812] | 7325 | -0.104 | 0.1212 | No |
| 39 | DPAGT1 | dolichyl-phosphate N-acetylglucosaminephosphotransferase 1 [Source:HGNC Symbol;Acc:HGNC:2995] | 7452 | -0.115 | 0.1184 | No |
| 40 | MPDU1 | mannose-P-dolichol utilization defect 1 [Source:HGNC Symbol;Acc:HGNC:7207] | 8330 | -0.180 | 0.0610 | No |
| 41 | GAL3ST1 | galactose-3-O-sulfotransferase 1 [Source:HGNC Symbol;Acc:HGNC:24240] | 8378 | -0.184 | 0.0685 | No |
| 42 | UGGT2 | UDP-glucose glycoprotein glucosyltransferase 2 [Source:HGNC Symbol;Acc:HGNC:15664] | 8568 | -0.199 | 0.0658 | No |
| 43 | FUT5 | fucosyltransferase 5 [Source:HGNC Symbol;Acc:HGNC:4016] | 8898 | -0.225 | 0.0538 | No |
| 44 | MGAT3 | "beta-1,4-mannosyl-glycoprotein 4-beta-N-acetylglucosaminyltransferase [Source:HGNC Symbol;Acc:HGNC:7046]" | 8920 | -0.226 | 0.0658 | No |
| 45 | PMM2 | phosphomannomutase 2 [Source:HGNC Symbol;Acc:HGNC:9115] | 9408 | -0.265 | 0.0440 | No |
| 46 | ENTPD5 | ectonucleoside triphosphate diphosphohydrolase 5 (inactive) [Source:HGNC Symbol;Acc:HGNC:3367] | 9414 | -0.265 | 0.0596 | No |
| 47 | FUT9 | fucosyltransferase 9 [Source:HGNC Symbol;Acc:HGNC:4020] | 9433 | -0.268 | 0.0744 | No |
| 48 | UGGT1 | UDP-glucose glycoprotein glucosyltransferase 1 [Source:HGNC Symbol;Acc:HGNC:15663] | 9516 | -0.275 | 0.0847 | No |
| 49 | FUT3 | fucosyltransferase 3 (Lewis blood group) [Source:HGNC Symbol;Acc:HGNC:4014] | 9890 | -0.311 | 0.0745 | No |
| 50 | MOGS | mannosyl-oligosaccharide glucosidase [Source:HGNC Symbol;Acc:HGNC:24862] | 10630 | -0.386 | 0.0403 | No |
| 51 | B4GALT1 | "beta-1,4-galactosyltransferase 1 [Source:HGNC Symbol;Acc:HGNC:924]" | 11530 | -0.505 | 0.0009 | No |
| 52 | TMEM165 | transmembrane protein 165 [Source:HGNC Symbol;Acc:HGNC:30760] | 11578 | -0.512 | 0.0282 | No |
| 53 | FUT6 | fucosyltransferase 6 [Source:HGNC Symbol;Acc:HGNC:4017] | 12067 | -0.612 | 0.0273 | No |
| 54 | ST3GAL1 | "ST3 beta-galactoside alpha-2,3-sialyltransferase 1 [Source:HGNC Symbol;Acc:HGNC:10862]" | 12092 | -0.619 | 0.0629 | No |
Table: GSEA details [plain text format]

  

Fig 2: GOBP\_PROTEIN\_N\_LINKED\_GLYCOSYLATION      
 Blue-Pink O' Gram in the Space of the Analyzed GeneSet

  

Fig 3: GOBP\_PROTEIN\_N\_LINKED\_GLYCOSYLATION: Random ES distribution      
 Gene set null distribution of ES for **GOBP\_PROTEIN\_N\_LINKED\_GLYCOSYLATION**
